# Complete steady-state rate equation for DNA ligase and its use for measuring product kinetic parameters of NAD^+^-dependent DNA ligase from *Haemophilus influenzae*

**DOI:** 10.1186/1756-0500-7-287

**Published:** 2014-05-09

**Authors:** Adam B Shapiro

**Affiliations:** 1 Biology Department, Infection Innovative Medicines Unit, AstraZeneca R&D Boston, 35 Gatehouse Drive, Waltham, MA 02451, USA

**Keywords:** DNA ligase, NAD^+^-dependent DNA ligase, Steady-state kinetics, Product inhibition, Bi Ter, Ping Pong, Kinetic mechanism

## Abstract

**Background:**

DNA ligase seals the nicks in the phosphodiester backbone between Okazaki fragments during DNA replication. DNA ligase has an unusual Bi Ter Ping Pong kinetic mechanism. Its substrates in eubacteria are NAD^+^ and nicked DNA (nDNA). Its products are nicotinamide mononucleotide (NMN), adenosine 5′-monophosphate (AMP), and sealed DNA. Investigation of the kinetic mechanism and measurement of the kinetic constants of DNA ligase using steady-state kinetics would benefit from the availability of the complete steady-state rate equation, including terms for product concentrations and product-related kinetic constants, which has not previously been published.

**Results:**

The rate equations for two possible Bi Ter kinetic mechanisms for DNA ligase, including products, are reported. The mechanisms differ according to whether the last two products, AMP and sealed DNA, are released in an ordered or rapid-equilibrium random (RER) manner. Steady-state kinetic studies of product inhibition by NMN and AMP were performed with *Haemophilus influenzae* NAD^+^-dependent DNA ligase. The complete rate equation enabled measurement of dissociation constants for NAD^+^, NMN, and AMP and eliminated one of 3 possible product release mechanisms.

**Conclusions:**

Steady-state kinetic product inhibition experiments and complete steady-state kinetic rate equations were used to measure dissociation constants of NAD^+^, NMN, and AMP and eliminate the possibility that AMP is the second product released in an ordered mechanism. Determining by steady-state kinetics whether the release of sealed DNA and AMP products goes by an ordered (AMP last off) or RER mechanism was shown to require a product inhibition study using sealed DNA.

## Findings

### Background

DNA ligase is the enzyme responsible for repairing nicks in the 5′-3′ phosphodiester bonds of the DNA backbone [1-3]. These nicks occur between the Okazaki fragments arising from lagging strand synthesis during DNA replication as well as DNA repair and recombination. In eukaryotes, archaea and some viruses, DNA ligases use ATP as the source of energy for the ligation reaction (E.C. 6.5.1.1), whereas eubacterial DNA ligases use NAD^+^ (E.C. 6.5.1.2), although additional ATP-dependent DNA ligases are found in some species of eubacteria. Because of this distinction, the essentiality of the enzyme for DNA replication, and the low sequence conservation between bacterial and mammalian DNA ligases, NAD^+^-dependent DNA ligase has recently been investigated as a target for novel antibacterial drug discovery [4-6].

DNA ligase has an unusual Bi Ter Ping Pong kinetic mechanism [7-9]. For NAD^+^-dependent DNA ligase, the 2 substrates are nicked DNA (nDNA) and NAD^+^. The 3 products are sealed DNA, adenosine 5′-monophosphate (AMP), and nicotinamide mononucleotide (NMN). For ATP-dependent DNA ligase, the products arising from ATP are AMP and inorganic pyrophosphate (PP_i_). Nick ligation involves 3 sequential reactions. In the 1^st^ step for NAD^+^-dependent DNA ligase, the enzyme reacts with NAD^+^, resulting in adenylylation of an active site lysine residue and release of NMN. In the 2^nd^ step, nDNA binds to the adenylylated enzyme and the AMP moiety is transferred to the 5′-phosphate of the nick. In the 3^rd^ step, the nick is repaired when the 3-OH group of the nick attacks the adenylylated 5′-phosphate, producing sealed DNA and AMP.

**Figure 1:**
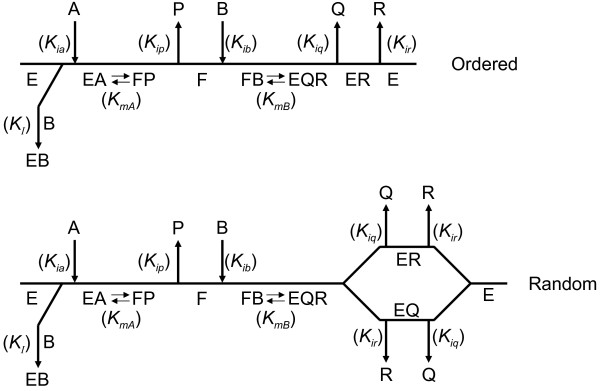
**Two possible Bi Ter Ping Pong Uni-Uni Uni-Bi kinetic mechanisms for DNA ligase. E** represents the enzyme with no ligands. **EA** represents the enzyme with NAD^+^**(A)** bound. **EB** represents the substrate-inhibited enzyme with nicked DNA **(B)** bound. **F** represents the adenylylated enzyme. **FP** represents the adenylylated enzyme with NMN **(P)** bound. **FB** represents the adenylyated enzyme with nicked DNA bound. **EQR** represents the enzyme with sealed DNA **(Q)** and AMP **(R)** bound. **EQ** represents the enzyme with sealed DNA bound. **ER** represents enzyme with AMP bound noncovalently. The mechanism in which the release of sealed DNA and AMP is ordered is shown at the top. The mechanism in which the order of release of sealed DNA and AMP is random is shown at the bottom. Kinetic constants relevant to each binding, dissociation, or catalytic step are shown in italics. Since no substrates bind and no products dissociate after the 2^nd^ step and before the 3^rd^ step of the reaction, the two steps are combined into FB↔EQR.

Although the 3-step kinetic mechanism of DNA ligase has long been known, the order of release of the last 2 products, sealed DNA and AMP, has rarely been investigated. Steady-state kinetic studies using product inhibition of bacteriophage T4 DNA ligase by AMP and PP_i_[9] contributed to the conclusion that the enzyme has a Ping Pong mechanism and is non-processive (i.e. it dissociates from DNA with each catalytic cycle rather than moving along it). Teraoka et al. [8] concluded from AMP and PP_i_ product inhibition studies of calf thymus DNA ligase that sealed DNA is released before AMP (Figure 1, *top*). It should be noted, however, that the authors did not address the possibility that the release of sealed DNA and AMP might be random (Figure 1, *bottom*) instead of ordered. Product inhibition by sealed DNA, which could have addressed this question, could not be investigated because no inhibition by sealed DNA was observed. Cooper and Rudolph [10], discussing the results of Teraoka et al. [8], pointed out that ordered release of sealed DNA then AMP would prevent product inhibition by sealed DNA within cells as long as the AMP concentration is low relative to its *K*_*I*_. Other investigators have measured the potency of inhibition of DNA ligases by AMP [11,12] or its binding affinity [13]. In no case, however, has the steady-state rate equation including product concentrations been published for the Bi Ter Ping Pong kinetic mechanism of DNA ligase.

In this paper, we derive the complete steady-state kinetic rate equations, including product terms, for the Bi Ter Ping Pong Uni-Uni Uni-Bi mechanisms in which the last 2 products are released in either an ordered or RER fashion (Additional file 1). We apply these equations to separate product inhibition studies with NMN and AMP to measure dissociation constants for NAD^+^, NMN, and AMP in the reaction of the NAD^+^-dependent DNA ligase from *Haemophilus influenza*. These studies allowed us to eliminate one of the 3 possible kinetic mechanisms for release of the sealed DNA and AMP products by this enzyme, namely release of AMP before sealed DNA. The complete rate equations demonstrate that a product inhibition study with sealed DNA, which was not feasible with our experimental system, would be required to determine by steady-state kinetics whether AMP is released after sealed DNA in an ordered mechanism or the two products are released in random order.

## Methods

### Materials

DNA ligase from *H. influenza* was purified as described previously [14]. DNA oligonucleotides were obtained from Eurofins MWG Operon (Huntsville, AL) or TriLink Biotechnologies (San Diego, CA). NAD^+^, NMN, AMP and reaction buffer components were from Sigma-Aldrich (St. Louis, MO).

### Derivations

The derivations of complete steady-state kinetic equations in Additional file 1 followed the method described by Segel [15] Ch. 9. The King-Altman analysis was performed with the program REFERASS [16].

### Product inhibition studies

The fluorescence resonance energy transfer assay used for enzyme kinetic assays was described in Shapiro et al. [17] and used the same DNA oligonucleotides. Assays were performed in 96-well, flat-bottom black polystyrene plates (Greiner Bio-One, Monroe, NC). Each combination of substrate, product, and enzyme concentration was present in a single well. The enzyme concentration was varied with the nDNA concentration so that each reaction would remain in the initial velocity range of product formation (fraction ligated ≤ 0.35). The DNA ligase concentrations were 72, 90, 108, 125, 160, and 200 pM with 10, 20, 30, 40, 60, and 80 nM nDNA, respectively. A separate plate was used for each product concentration. Reactions were performed at room temperature for 30 min in a volume of 100 μl, quenched with 100 μl of quench solution, and incubated for 20 min at room temperature. Fluorescence emission intensities were measured at 535 and 595 nm upon excitation at 485 nm with a TECAN Ultra plate reader (BMG Labtech, Cary, NC). After subtraction of background fluorescence in the absence of nDNA at each NAD^+^ concentration, the ratios of 595 to 535 nm fluorescence intensities were calculated. The ratio change (ΔRatio) was calculated by subtracting the ratio at 0 μM NAD^+^ for each nDNA concentration. ΔRatio values were converted into fraction of DNA ligated using the method described in Shapiro et al. [17]. The values of the constants **a** and **b**[17] were 0.345 and 0.706, respectively. Values of fraction ligated were converted into specific activities based on the nDNA and enzyme concentrations. Data were analysed and graphed with Grafit (Erithacus Software, Horley, Surrey, UK). The product inhibition studies used non-linear least squares analysis rather than Lineweaver-Burk double-reciprocal plots to fit the experimental data to steady-state rate equations. The former method was preferred because the potent substrate inhibition caused by nicked DNA [K_i_ ≈ K_m_(nDNA)] in our experimental system results in double reciprocal plots that are curvilinear and therefore not well-suited to interpretation.

## Results

### Complete Bi Ter Ping Pong rate equations

The complete steady-state kinetic rate equations for the Bi Ter Ping Pong mechanisms shown in Figure 1 are derived in Additional file 1 and are shown below (Den is short for denominator).

$$V=\frac{\mathrm{Vmax},f\left[A\right]\left[B\right]-\mathrm{Vmax},f\left[P\right]\left[Q\right]\left[R\right]/\mathrm{Keq}}{\mathrm{Den}}$$

where

Den = K_mB_[A] + K_mA_[B] + (K_mA_/K_I_)[B]^2^ + [A][B] + (K_ia_K_mB_/K_ip_)[P] + (K_mb_/K_ip_)[A][P] +

{K_mB_K_mR_/(K_mQ_K_ir_)}[A][Q] + {K_ia_K_mB_K_mR_/(K_ip_K_mQ_K_ir_)}[P][Q] +

{K_ia_K_mB_/(K_ip_K_ir_)}[P][R] + (K_mA_/K_ir_)[B][R] + {K_mA_K_ib_/(K_iq_K_ir_)}[Q][R] +

{K_mB_K_mR_/(K_ib_K_mQ_K_ir_)}[A][B][Q] + {K_mR_K_mB_/(K_ip_K_mQ_K_ir_)}[A][P][Q] +

{K_ia_K_mB_/(K_ip_K_mQ_K_ir_)}[P][Q][R] + {K_mA_/(K_iq_K_ir_)}[B][Q][R] +

{K_ia_K_mB_/(K_ip_K_I_)}[B][P] + {K_ia_K_mB_K_mR_/(K_ip_K_mQ_K_ir_K_I_)}[B][P][Q]

for the case where release of the last two products is ordered,

or where

Den = K_mB_[A] + K_mA_[B] + (K_mA_/K_I_)[B]^2^ + [A][B] + (K_ia_K_mB_/K_ip_)[P] +

{K_ia_K_mB_/(K_ip_K_I_)}[B][P] + (K_mb_/K_ip_)[A][P] +

{K_ia_K_mB_/(K_ip_K_iq_)}[P][Q] + {K_ia_K_mB_/(K_ip_K_iq_K_I_)}[B][P][Q] + (K_mA_/K_iq_)[B][Q] + {K_mA_K_ib_/(K_iq_K_ir_)}[Q][R] + {K_ia_K_mB_/(K_ip_K_ir_)}[P][R] + (K_mA_/K_ir_)[B][R] +

{K_ia_K_mB_/(K_ip_K_mQ_K_ir_)}[P][Q][R] + {K_mA_/(K_iq_K_ir_)}[B][Q][R]

for the case where release of the last two products goes by an RER mechanism.

If no more than two of the products are present at the start of the reaction, then

V_max,f_[P][Q][R]/K_eq_ = 0 so that V = V_max,f_[A][B]/Den for the initial rate in the forward direction.

For NAD^+^-dependent DNA ligase, A is NAD^+^, B is nDNA, and P is NMN. There are 3 possible mechanisms for the release of Q and R, which are sealed DNA and AMP in an unspecified order.

i. An ordered release in which Q is AMP and R is sealed DNA.

ii. An ordered release in which Q is sealed DNA and R is AMP.

iii. A random release of Q and R, in which case it is arbitrary whether Q is AMP and R is sealed DNA, or Q is sealed DNA and R is AMP.

### Product inhibition by NMN

For product inhibition studies by only NMN, the first product (P), the two mechanisms have the same rate equation, in which:

$$\begin{array}{l} \mathrm{Den}=K_{\mathrm{mB}}\left[A\right]\left(1+\left[P\right]/K_{\mathrm{ip}}\right)+K_{\mathrm{mA}}\left[B\right]\left(1+\left[B\right]/K_{I}\right)\\ \;+\left[A\right]\left[B\right]+\left(K_{\mathrm{ia}}K_{\mathrm{mB}}/K_{\mathrm{ip}}\right)\left[P\right]\left(1+\left[B\right]/K_{I}\right). \end{array}$$

Thus a product inhibition study with NMN provides values for K_ia_, which is the dissociation constant of NAD^+^ from unadenylylated DNA ligase, and K_ip_, which is the dissociation constant of the product NMN from adenylylated DNA ligase.

**Figure 2:**
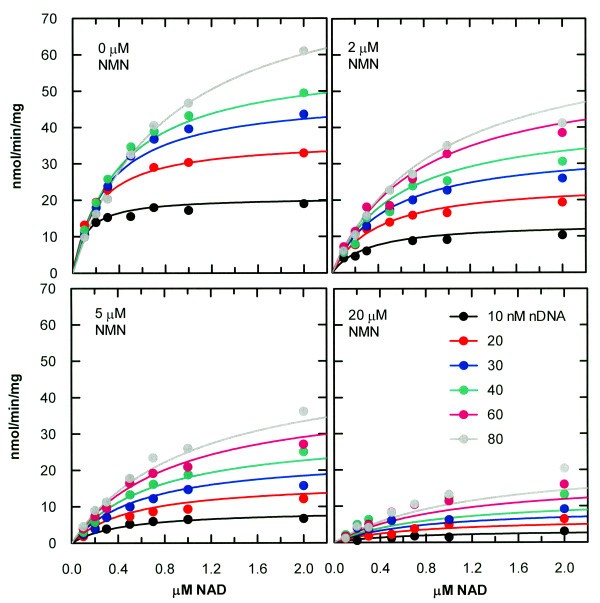
**Global non-linear least-squares fit of data from NMN product inhibition of *H. influenzae *DNA ligase.** The data were fit to the Bi Ter Ping Pong Uni-Uni Uni-Bi steady-state rate equation with product P present: *V* = *V*_*max*_[*A*][*B*]/(*K*_*mB*_[*A*] + *K*_*mA*_[*B*](1 + [*B*]/*K*_*I*_) + [*A*][*B*] + (*K*_*ia*_*K*_*mB*_/*K*_*ip*_)[*P*] + (*K*_*mB*_/*K*_*ip*_)[*A*][*P*] + {*K*_*ia*_*K*_*mB*_/(*K*_*ip*_*K*_*I*_)}[*B*][*P*]). Each data point represents a single measurement. The 60 nM DNA data at 0 mM AMP were omitted due to poor quality.

The results of a product inhibition study with NMN are shown in Figure 2 and Table 1, with best-fit curves based on the expression for Den above. Except for a few minor deviations and a slight underestimation of activity with the higher nDNA concentrations at 20 μM NMN, the data are very well-fit by the model. The values of V_max_, K_mA_ (the Michaelis constant for NAD^+^) and K_mB_ (the Michaelis constant for nDNA) are consistent with the values reported previously [17]. The values of K_ia_ and K_mA_ are approximately equal at about 0.6 μM. The dissociation constant for the product NMN is only about 5-fold higher than that of the substrate NAD^+^. This situation is unlikely to cause substantial product inhibition in vivo, however, because the NAD^+^ concentration in the cytoplasm is vastly higher than the K_m_(NAD^+^). Bennett et al. [18] reported the cytoplasmic NAD^+^ concentration of *E. coli* to be approximately 2.6 mM.

**Table 1:** **Kinetic parameters obtained by nonlinear least-squares global fitting of data from Figures**2**and**3

**Parameter**	**Value from NMN study**	**Value from AMP study**
V_max_	150 ± 15 nmol/min/mg	169 ± 13 nmol/min/mg
K_mA_	650 ± 80 nM	410 ± 50 nM
K_mB_	62 ± 9 nM	85 ± 9 nM
K_i_	66 ± 14 nM	32 ± 8 nM
K_ia_	610 ± 120 nM	---
K_ip_	3.3 ± 0.4 μM	---
K_ir_	---	390 ± 60 μM

K_mA_ and K_mB_ are the Michaelis constants for NAD^+^ and nDNA, respectively. K_i_ is the dissociation constant for nDNA with unadenylylated DNA ligase (i.e. substrate inhibition constant for nDNA). K_ia_ is the dissociation constant for NAD^+^ with unadenylylated DNA ligase. K_ip_ is the dissocation constant for NMN. K_ir_ is the dissociation constant for AMP. Standard errors of best-fit values are shown.

### Product inhibition by AMP

For product inhibition by only AMP, the steady-state rate equation depends upon the mechanism of product release. For RER release of AMP and sealed DNA, if AMP is product R (or equivalently product Q), or for ordered release if AMP is product R (i.e. sealed DNA is released before AMP), then

$$\begin{array}{l} \mathrm{Den}=K_{\mathrm{mB}}\left[A\right]+K_{\mathrm{mA}}\left[B\right]\left(1+\left[B\right]/K_{I}+\left[R\right]/K_{\mathrm{ir}}\right)+\left[A\right]\\ \;\times \left[B\right]. \end{array}$$

For ordered release of AMP and sealed DNA, if AMP is product Q (i.e. if AMP is released before sealed DNA), then

$$\begin{array}{l} \mathrm{Den}=K_{\mathrm{mB}}\left[A\right]\left\{1+K_{\mathrm{mR}}/\left(K_{\mathrm{mQ}}K_{\mathrm{ir}}\right)\left[Q\right]\;\left(1+\left[B\right]/K_{\mathrm{ib}}\right)\right\}\\ \;+K_{\mathrm{mA}}\left[B\right]\left(1+\left[B\right]/K_{I}\right)+\left[A\right]\left[B\right]. \end{array}$$

**Figure 3:**
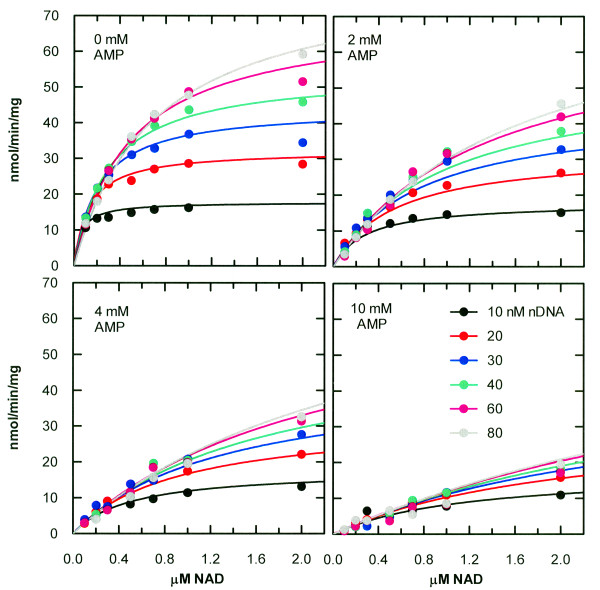
**Global non-linear least-squares fit of data from AMP product inhibition of *H. influenzae *DNA ligase.** The data were fit to the Bi Ter Ping Pong Uni-Uni Uni-Bi steady-state rate equation with product R present: *V* = *V*_*max*_[*A*][*B*]/(*K*_*mB*_[*A*] + *K*_*mA*_[*B*](1 + [*B*]/*K*_*I*_ + [*R*]/*K*_*ir*_) + [*A*][*B*]) Each data point represents a single measurement.

A product inhibition experiment with AMP should be able to distinguish between the two cases. The results of such a study are shown in Figure 3. The global fitting procedure was not able to identify a meaningful set of parameter values for the second case, ordered release of AMP followed by sealed DNA. [Note that K_mR_/(K_mQ_K_ir_) was fit as a single combined parameter]. For the first case, a high-quality fit was readily achieved. The best-fit values are given in Table 1. This experiment provided a value for K_ir_, the dissociation constant of AMP, of about 400 μM. The ordered mechanism in which AMP is released before sealed DNA was not consistent with the data. Thus, either AMP is released last in an ordered release of sealed DNA and AMP or sealed DNA and AMP are released in a RER mechanism.

## Discussion

The availability of the complete steady-state kinetic rate equations for the Bi Ter Ping Pong mechanism of DNA ligase and a quantitative high-throughput FRET assay made it possible to use product inhibition studies with NMN and AMP to obtain 3 dissociation constants; K_ia_, K_ip_, and K_ir_ for NAD^+^, NMN, and AMP, respectively; that could not be obtained by steady-state kinetics with substrates only. In addition, these equations and the product inhibition studies made it possible to eliminate from consideration one of the 3 possible kinetic mechanisms for release of the last 2 products, sealed DNA and AMP. The same approach could be used for ATP-dependent DNA ligases, substituting ATP for NAD^+^ and PP_i_ for NMN.

To distinguish between the remaining 2 possible kinetic mechanisms for release of sealed DNA and AMP (and to obtain a value for K_iq_ if the mechanism were RER) by steady-state kinetics would require a product inhibition experiment with sealed DNA. This experiment was not possible with the DNA ligase FRET assay, which detects sealed DNA. A different assay format would be required to perform that experiment. Even with a suitable assay, however, the experiment may be impractical if there is insufficient inhibition by sealed DNA. Teraoka et al. [8] observed no inhibition of calf thymus ATP-dependent DNA ligase by sealed DNA. It is reasonable to expect that the affinity of DNA ligase for sealed DNA at any step in its catalytic mechanism would be negligible considering the enormous amount of nick-free DNA present in the bacterial cytoplasm or eukaryotic nucleus. Otherwise, DNA ligase could experience severe product inhibition by the sealed DNA.

The cytoplasmic AMP concentration of approximately 300 μM reported by Bennett et al. [18] for *E. coli* grown with glucose is similar to the AMP dissociation constant K_ir_ of *H. influenzae* DNA ligase, measured here at 390 ± 60 μM. Assuming the *H. influenzae* cytoplasmic AMP concentration to be similar to that of *E. coli*, if AMP is product R (i.e. released last) in an ordered release mechanism, or if AMP and sealed DNA dissociate by a RER mechanism, product inhibition by AMP would be significant in vivo if it were not for the fact that the NAD^+^ concentration is so high compared to K_m_(NAD^+^). AMP acts as a dead-end inhibitor with respect to NAD^+^ according to these mechanisms, so the saturating NAD^+^ concentration would prevent product inhibition by AMP. Similarly, the high ratio of cytoplasmic NAD^+^ concentration relative to its K_m_ minimizes substrate inhibition by nDNA.

The suggestion by Cooper and Rudolph [10] that ordered release of sealed DNA then AMP would prevent product inhibition by sealed DNA within cells as long as the AMP concentration is low relative to its *K*_*I*_ does not apply in the case of *H. influenzae* NAD^+^-dependent DNA ligase because the *K*_*I*_ for AMP is likely to be similar to the cytoplasmic AMP concentration. Therefore, it is more likely that product inhibition by sealed DNA is prevented by a lack of affinity of the enzyme for sealed DNA. This means of preventing inhibition by sealed DNA would also be effective if sealed DNA and AMP were released by an RER mechanism.

## Conclusions

By means of product inhibition studies of *H. influenza* DNA ligase with AMP and NMN and newly derived complete steady-state kinetic rate equations including products, the dissociation constants for the substrate NAD^+^ and the products NMN and AMP were measured. The studies also eliminated one of 3 possible kinetic mechanisms for product release, namely that AMP is release before sealed DNA in an ordered mechanism. The 2 remaining possible mechanisms are (1) sealed DNA is released before AMP in an ordered mechanism or (2) the release of sealed DNA and AMP are random and in rapid equilibrium.

## Competing interests

The author is an employee of AstraZeneca.

## Supplementary Material

Additional file 1 Derivation of complete rate equations for Bi Ter Ping Pong Uni-Uni Uni-Bi kinetic mechanisms. Click here for file
