# Supplementary material for: Complete steady-state rate equation for DNA ligase and its use for measuring product kinetic parameters of NAD+-dependent DNA ligase from Haemophilus influenzae
Source: BMC Res Notes. 2014 May 9;7:287. doi: 10.1186/1756-0500-7-287 (PMC4022432; doi:10.1186/1756-0500-7-287)
Supplement: Additional file 1 — Derivation of complete rate equations for Bi Ter Ping Pong Uni-Uni Uni-Bi kinetic mechanisms. [file 1756-0500-7-287-S1.docx]

Appendix 1. Derivation of complete rate equation for Bi Ter Ping Pong Uni-Uni Uni-Bi kinetic mechanism with substrate inhibition by substrate B and ordered release of products Q and R.

**Part 1. REFERASS input**

Names of species

X1 = E

X2 = EA/FP

X3 = F

X4 = FB/EQR

X5 = ER

X6 = EB

Constants

K1,2 = k+1[A]

K2,1 = k-1

K2,3 = k+2

K3,2 = k-2[P]

K3,4 = k+3[B]

K4,3 = k-3

K4,5 = k+4

K5,4 = k-4[Q]

K5,1 = k+5

K1,5 = k-5[R]

K1,6 = k+6[B]

K6,1 = k-6

**Part 2. REFERASS output**

Distribution equations

[E] = N(E)[E]o/Den

[EA/FP] = N(EA/FP)[E]o/Den

[F] = N(F)[E]o/Den

[FB/EQR] = N(FB/EQR)[E]o/Den

[ER] = N(ER)[E]o/Den

[EB] = N(EB)[E]o/Den

Definitions

(Note: K_6_ = k_-6_/k_+6_)

N(E) = k_-1_k_-2_k_+5_K_6_(k_-3_ + k_+4_}[P] + k_+3_k_+4_k_+5_K_6_(k_-1_ + k_+2_)[B] + k_-1_k_-2_k_-3_k_-4_K_6_[P][Q]

N(EA/FP) = k_+1_k_-2_k_+5_K_6_(k_-3_ + k_+4_)[A][P] + k_+1_k_+3_k_+4_k_+5_K_6_[A][B] + k_+1_k_-2_k_-3_k_-4_K_6_[A][P][Q]

+ k_-2_k_-3_k_-4_k_-5_K_6_[P][Q][R]

N(F) = k_+1_k_+2_k_+5_K_6_(k_-3_ + k_+4_)[A] + k_+1_k_+2_k_-3_k_-4_K_6_[A][Q] + k_-3_k_-4_k_-5_K_6_(k_-1_ + k_+2_)[Q][R]

N(FB/EQR) = k_+1_k_+2_k_+3_k_+5_K_6_[A][B] + k_+1_k_+2_k_+3_k_-4_K_6_[A][B][Q] + k_-1_k_-2_k_-4_k_-5_K_6_[P][Q][R]

+ k_+3_k_-4_k_-5_K_6_(k_-1_ + k_+2_)[B][Q][R]

N(ER) = k_+1_k_+2_k_+3_k_+4_K_6_[A][B] + k_-1_k_-2_k_-5_K_6_(k_-3_ + k_+4_)[P][R] + k_+3_k_+4_k_-5_K_6_(k_-1_ + k_+2_)[B][R]

N(EB) = k_-1_k_-2_k_+5_(k_-3_ + k_+4_)[B][P] + k_+3_k_+4_k_+5_(k_-1_ + k_+2_)[B]^2 + k_-1_k_-2_k_-3_k_-4_[B][P][Q]

Den = k_+1_k_+2_k_+5_K_6_(k_-3_ + k_+4_)[A] + k_-1_k_-2_k_+5_K_6_(k_-3_ + k_+4_)[P] + k_+3_k_+4_k_+5_K_6_(k_-1_ + k_+2_)[B] +

k_+1_k_+3_K_6_(k_+2_k_+4_ + k_+2_k_+5_ + k_+4_k_+5_)[A][B] + k_+1_k_+2_k_-3_k_-4_K_6_[A][Q] +

k_+1_k_-2_k_+5_K_6_(k_-3_ + k_+4_)[A][P] + k_-1_k_-2_k_-3_k_-4_K_6_[P][Q] + k_-1_k_-2_k_-5_K_6_(k_-3_ + k_+4_)[P][R] +

k_+3_k_+4_k_-5_K_6_(k_-1_ + k_+2_)[B][R] + k_-3_k_-4_k_-5_K_6_(k_-1_ + k_+2_)[Q][R] + k_-1_k_-2_k_+5_(k_-3_ + k_+4_)[B][P]

+ k_+3_k_+4_k_+5_(k_-1_ + k_+2_)[B]^2 + k_+1_k_+2_k_+3_k_-4_K_6_[A][B][Q] + k_+1_k_-2_k_-3_k_-4_K_6_[A][P][Q] +

k_-2_k_-4_k_-5_K_6_(k_-1_ + k_-3_)[P][Q][R] + k_+3_k_-4_k_-5_K_6_(k_-1_ + k_+2_)[B][Q][R] + k_-1_k_-2_k_-3_k_-4_[B][P][Q]

M(P) = M(Q) = M(R) = k_+1_k_+2_k_+3_k_+4_k_+5_K_6_[A][B] – k_-1_k_-2_k_-3_k_-4_k_-5_K_6_[P][Q][R]

**Part 3. Coefficients**

Coef_A_ = k_+1_k_+2_k_+5_K_6_(k_-3_ + k_+4_)

Coef_P_ = k_-1_k_-2_k_+5_K_6_(k_-3_ + k_+4_)

Coef_B_ = k_+3_k_+4_k_+5_K_6_(k_-1_ + k_+2_)

Coef_AB_ = k_+1_k_+3_K_6_(k_+2_k_+4_ + k_+2_k_+5_ + k_+4_k_+5_)

Coef_AQ_ = k_+1_k_+2_k_-3_k_-4_K_6_

Coef_AP_ = k_+1_k_-2_k_+5_K_6_(k_-3_ + k_+4_)

Coef_PQ_ = k_-1_k_-2_k_-3_k_-4_K_6_

Coef_PR_ = k_-1_k_-2_k_-5_K_6_(k_-3_ + k_+4_)

Coef_BR_ = k_+3_k_+4_k_-5_K_6_(k_-1_ + k_+2_)

Coef_QR_ = k_-3_k_-4_k_-5_K_6_(k_-1_ + k_+2_)

Coef_BP_ = k_-1_k_-2_k_+5_(k_-3_ + k_+4_)

Coef_B_^2^ = k_+3_k_+4_k_+5_(k_-1_ + k_+2_)

Coef_ABQ_ = k_+1_k_+2_k_+3_k_-4_K_6_

Coef_APQ_ = k_+1_k_-2_k_-3_k_-4_K_6_

Coef_PQR_ = k_-2_k_-4_k_-5_K_6_(k_-1_ + k_-3_)

Coef_BQR_ = k_+3_k_-4_k_-5_K_6_(k_-1_ + k_+2_)

Coef_BPQ_ = k_-1_k_-2_k_-3_k_-4_

**Part 4. Kinetic constants**

num1 = k_+1_k_+2_k_+3_k_+4_k_+5_K_6_[E]_t_

num2 = k_-1_k_-2_k_-3_k_-4_k_-5_K_6_[E]_t_

K_eq_ = num1/num2

Vmax_f_ = num1/Coef_AB_ = k_+2_k_+4_k_+5_[E]_t_/(k_+2_k_+4_ + k_+2_k_+5_ + k_+4_k_+5_)

Vmax_r_ = num2/Coef_PQR_ = k_-1_k_-3_[E]_t_/(k_-1_ + k_-3_)

K_mA_ = Coef_B_/Coef_AB_ = k_+4_k_+5_(k_-1_ + k_+2_)/[k_+1_(k_+2_k_+4_ + k_+2_k_+5_ + k_+4_k_+5_)]

K_mB_ = Coef_A_/Coef_AB_ = k_+2_k_+5_(k_-3_ + k_+4_)/[k_+3_(k_+2_k_+4_ + k_+2_k_+5_ + k_+4_k_+5_)]

K_mP_ = Coef_QR_/Coef_PQR_ = k_-3_(k_-1_ + k_+2_)/[k_-2_(k_-1_ + k_-3_)]

K_mQ_ = Coef_PR_/Coef_PQR_ = k_-1_(k_-3_ + k_+4_)/[k_-4_(k_-1_ + k_-3_)]

K_mR_ = Coef_PQ_/Coef_PQR_ = k_-1_k_-3_/[k_-5_(k_-1_ + k_-3_)]

K_ia_ = Coef_P_/Coef_AP_ = Coef_PQ_/Coef_APQ_ = k_-1_/k_+1_

K_ib_ = Coef_AQ_/Coef_ABQ_ = Coef_QR_/Coef_BQR_ = k_-3_/k_+3_

K_ip_ = Coef_A_/Coef_AP_ = Coef_AQ_/Coef_APQ_ = k_+2_/k_-2_

K_iq_ = Coef_BR_/Coef_BQR_ = k_+4_/k_-4_

K_ir_ = Coef_P_/Coef_PR_ = Coef_B_/Coef_BR_ = k_+5_/k_-5_

K_I_ = Coef_P_/Coef_BP_ = Coef_B_/Coef_B^2_ = Coef_PQ_/Coef_BPQ_ = K_6_ = k_-6_/k_+6_

**Part 5. Haldane relationship**

Vmax_f_/(Vmax_r_K_eq)_ = K_ia_K_mB_/(K_ip_K_mQ_K_ir_) = K_mA_K_ib_/(K_mP_K_iq_K_ir_) =

k_-2_k_-4_k_-5_(k_-1_ + k_-3_)/[k_+1_k_+3_(k_+2_k_+4_ + k_+2_k_+5_ + k_+4_k_+5_)]

**Part 6. Complete rate equation**

V = (num1[A][B] – num2[P][Q][R])/Den

Den = Coef_A_[A] + Coef_P_[P] + Coef_B_[B] + Coef_AB_[A][B] + Coef_AQ_[A][Q] + Coef_AP_[A][P] + Coef_PQ_[P][Q] + Coef_PR_[P][R] + Coef_BR_[B][R] + Coef_QR_[Q][R] + Coef_ABQ_[A][B][Q] + Coef_APQ_[A][P][Q] + Coef_PQR_[P][Q][R] + Coef_BQR_[B][Q][R] + + Coef_BP_[B][P] + Coef_B^2_[B]^2 + Coef_BPQ_[B][P][Q]

Multiply numerator and denominator by num2/(Coef_AB_Coef_PQR_) or, equivalently, num1num2/(num1Coef_AB_Coef_PQR_) as needed, where num1num2/(num1Coef_AB_) = Vmax_f_/K_eq_

V = {num1num2[A][B]/(Coef_AB_Coef_PQR_) –

num1num2num2[P][Q][R]/(num1Coef_AB_Coef_PQR_)}/Den =

{Vmax_f_Vmax_r_[A][B] – Vmax_f_Vmax_r_[P][Q][R]/K_eq_}/Den

Substitute kinetic constants for coefficients in denominator, as follows:

Coef_A_: num2Coef_A_/(Coef_AB_Coef_PQR_) = K_mB_Vmax_r_

Coef_B_: num2Coef_B_/(Coef_AB_Coef_PQR_) = Vmax_r_Km_A_

Coef_B_^2^: num2Coef_B_^2^/(Coef_AB_Coef_PQR_) = Vmax_r_K_mA_/K_I_

Coef_AB_: num2Coef_AB_/(Coef_AB_Coef_PQR_) = Vmax_r_

Coef_P_: num2Coef_P_/(Coef_AB_Coef_PQR_) = Vmax_r_K_ia_Coef_AP_/(Coef_AB_) =

Vmax_r_K_ia_Coef_A_/(K_ip_Coef_AB_) = Vmax_r_K_ia_K_mB_/K_ip_

Coef_AP_: num2Coef_AP_/(Coef_AB_Coef_PQR_) = num2Coef_A_/(K_ip_Coef_AB_Coef_PQR_) = Vmax_r_K_mB_/K_ip_

Coef_AQ_: num1num2Coef_AQ_/(num1Coef_AB_Coef_PQR_) = Vmax_f_K_ip_Coef_APQ_/(K_eq_Coef_PQR_) =

Vmax_f_K_ip_Coef_PQ_/(K_eq_Coef_PQR_K_ia_) = Vmax_f_K_ip_K_mR_/(K_eq_K_ia_)

Coef_PQ_: num1num2Coef_PQ_/(num1Coef_AB_Coef_PQR_) = Vmax_f_K_mR_/K_eq_

Coef_PR_: num1num2Coef_PR_/(num1Coef_AB_Coef_PQR_) = Vmax_f_K_mQ_/K_eq_

Coef_BR_: num2Coef_BR_/(Coef_AB_Coef_PQR_) = Vmax_r_Coef_B_/(Coef_AB_K_ir_) = Vmax_r_K_mA_/K_ir_

Coef_QR_: num1num2Coef_QR_/(num1Coef_AB_Coef_PQR_) = Vmax_f_K_mP_/K_eq_

Coef_ABQ_: num1num2Coef_ABQ_/(num1Coef_AB_Coef_PQR_) = Vmax_f_Coef_AQ_/(K_eq_K_ib_Coef_PQR_) =

Vmax_f_K_ip_Coef_APQ_/(K_eq_K_ib_Coef_PQR_) = Vmax_f_K_ip_Coef_PQ_/(K_eq_K_ia_K_ib_Coef_PQR_) =

Vmax_f_K_ip_K_mR_/(K_eq_K_ia_K_ib_)

Coef_APQ_: num1num2Coef_APQ_/(num1Coef_AB_Coef_PQR_) = Vmax_f_Coef_PQ_/(K_eq_K_ia_Coef_PQR_) =

Vmax_f_K_mR_/(K_eq_K_ia_)

Coef_PQR_: num1num2Coef_PQR_/(num1Coef_AB_Coef_PQR_) = Vmax_f_/K_eq_

Coef_BQR_: num1num2Coef_BQR_/(num1Coef_AB_Coef_PQR_) = Vmax_f_Coef_QR_/(K_eq_K_ib_Coef_PQR_) =

Vmax_f_K_mP_/(K_eq_K_ib_)

Coef_BP_: num1num2Coef_BP_/(num1Coef_AB_Coef_PQR_) = Vmax_f_Coef_P_/(K_eq_K_I_Coef_PQR_) =

Vmax_f_K_ir_Coef_PR_/(K_eq_K_I_Coef_PQR_) = Vmax_f_K_mQ_K_ir_/(K_eq_K_I_)

Coef_BPQ_: num1num2Coef_BPQ_/(num1Coef_AB_Coef_PQR_) = Vmax_f_Coef_PQ_/(K_eq_K_I_Coef_PQR_) =

Vmax_f_K_mR_/(K_eq_K_I_)

Den = Vmax_r_K_mB_[A] + Vmax_f_K_mQ_K_ir_[P]/K_eq_ + Vmax_r_K_mA_[B] + Vmax_r_[A][B] +

Vmax_f_K_ip_K_mR_[A][Q]/(K_eq_K_ia_) + Vmax_r_K_mB_[A][P]/K_ip_ + Vmax_f_K_mR_[P][Q]/K_eq_+

Vmax_f_K_mQ_[P][R]/K_eq_ + Vmax_r_K_mA_[B][R]/K_ir_ + Vmax_f_K_mP_[Q][R]/K_eq_ +

Vmax_f_K_ip_K_mR_[A][B][Q]/(K_eq_K_ia_K_ib_) + Vmax_f_K_mR_[A][P][Q]/(K_eq_K_ia_) +

Vmax_f_[P]Q][R]/K_eq_ + Vmax_f_K_mP_[B][Q][R]/(K_eq_K_ib_) + Vmax_f_K_mQ_K_ir_[B][P]/(K_eq_K_I_) +

Vmax_r_K_mA_[B]^2^/K_I_ + Vmax_f_K_mR_[B][P][Q]/(K_eq_K_I_)

Divide numerator and denominator by Vmax_r_.

Substitute Haldane relationship:

Vmax_f_/(Vmax_r_K_eq_) = K_ia_K_mB_/(K_ip_K_mQ_K_ir_) = K_mA_K_ib_/(K_mP_K_iq_K_ir_)

V = {Vmax_f_[A][B] – Vmax_f_[P][Q][R]/K_eq_}/Den

Den = K_mB_[A] + K_mA_[B] + (K_mA_/K_I_)[B]^2^ + [A][B] + (K_ia_K_mB_/K_ip_)[P] + (K_mb_/K_ip_)[A][P] +

{K_mB_K_mR_/(K_mQ_K_ir_)}[A][Q] + {K_ia_K_mB_K_mR_/(K_ip_K_mQ_K_ir_)}[P][Q] +

{K_ia_K_mB_/(K_ip_K_ir_)}[P][R] + (K_mA_/K_ir_)[B][R] + {K_mA_K_ib_/(K_iq_K_ir_)}[Q][R] +

{K_mB_K_mR_/(K_ib_K_mQ_K_ir_)}[A][B][Q] + {K_mR_K_mB_/(K_ip_K_mQ_K_ir_)}[A][P][Q] +

{K_ia_K_mB_/(K_ip_K_mQ_K_ir_)}[P][Q][R] + {K_mA_/(K_iq_K_ir_)}[B][Q][R] +

{K_ia_K_mB_/(K_ip_K_I_)}[B][P] + {K_ia_K_mB_K_mR_/(K_ip_K_mQ_K_ir_K_I_)}[B][P][Q]

**Part 7. No- and one-product rate equations**

For measurements of initial rates when at least one product is absent, the numerator reduces to

Vmax_f_[A][B].

For measurements of initial rates with no products initially present, the rate equation reduces to:

V = Vmax[A][B]/{K_mB_[A] + K_mA_[B] + (K_mA_/K_I_)[B]^2^ + [A][B]} or

V = Vmax[A][B]/{K_mB_[A] + K_mA_[B](1 + [B]/K_I_) + [A][B]}

-----------------------------------------------------------------------------------------------------------------

For measurements of initial rates with only product P and substrates present, the denominator reduces to:

Den = K_mB_[A] + K_mA_[B](1 + [B]/K_I_) + [A][B] + (K_ia_K_mB_/K_ip_)[P] + (K_mB_/K_ip_)[A][P] +

{K_ia_K_mB_/(K_ip_K_I_)}[B][P]

Grouping the [P] terms gives:

Den = K_mB_[A] + K_mA_[B](1 + [B]/K_I_) + [A][B] + (K_ia_K_mB_/K_ip_)[P](1 + [A]/K_ia_ + [B]/K_I_) or

Den = K_mB_[A](1 + [P]/K_ip_) + K_mA_[B](1 + [B]/K_I_) + [A][B] + (K_ia_K_mB_/K_ip_)[P](1 + [B]/K_I_)

The latter arrangement of the equation shows that product P acts in part as a dead-end inhibitor by binding to the enzyme form F, represented in the equation by the term K_mB_[A], in competition with substrate B. The equation also shows that inhibition by product P and substrate inhibition by substrate B are interdependent, such that substrate inhibition by B reduces the effect of product inhibition by P.

-----------------------------------------------------------------------------------------------------------------

For measurements of initial rates with only product Q and substrates present, the denominator reduces to:

Den = K_mB_[A] + K_mA_[B](1 + [B]/K_I_)+ [A][B] +{K_mB_K_mR_/(K_mQ_K_ir_)}[A][Q] +

+{K_mB_K_mR_/(K_ib_K_mQ_K_ir_)}[A][B][Q] or

Den = K_mB_[A] + K_mA_[B](1 + [B]/K_I_)+ [A][B] +{K_mB_K_mR_/(K_mQ_K_ir_)}[A][Q](1+[B]/K_ib_) or

Den = K_mB_[A]{1 + K_mR_/(K_mQ_K_ir_)[Q] (1+[B]/K_ib_)} + K_mA_[B](1 + [B]/K_I_)+ [A][B]

The last arrangement of the equation shows that product Q acts as a dead-end inhibitor by binding to enzyme form F, represented by the term K_mB_[A], in competition with substrate B.

-----------------------------------------------------------------------------------------------------------------

For measurements of initial rates with only product R and substrates present, the denominator reduces to:

Den = K_mB_[A] + K_mA_[B](1 + [B]/K_I_) + [A][B] + (K_mA_/K_ir_)[B][R] or

Den = K_mB_[A] + K_mA_[B](1 + [B]/K_I_ + [R]/K_ir_) + [A][B]

The latter equation shows that product R acts as a dead-end inhibitor by binding to the unliganded enzyme E in a manner that is mutually exclusive with substrate inhibition by substrate B.

Appendix 2. Derivation of complete rate equation for Bi Ter Ping Pong Uni-Uni Uni-Bi kinetic mechanism with substrate inhibition by substrate B and rapid equilibrium random release of products Q and R.

The derivation of the rate equation was first performed without substrate inhibition according to the following mechanism.

**Part 1. REFERASS input**

Names of species

X1 = E

X2 = EA/FP

X3 = F

X4 = FB/EQR

X5 = EQ

X6 = ER

Constants

K1,2 = k+1[A]

K2,1 = k-1

K2,3 = k+2

K3,2 = k-2[P]

K3,4 = k+3[B]

K4,3 = k-3

K4,5 = k+4

K5,4 = k-4[R]

K4,6 = k+5

K6,4 = k-5[Q]

K5,1 = k+6

K1,5 = k-6[Q]

K6,1 = k+7

K1,6 = k-7[R]

RE 4,5

RE 5,1

RE 4,6

RE 6,1

**Part 2. REFERASS output**

Distribution equations

[E] = N(E)[E]o/Den

[EA/FP] = N(EA/FP)[E]o/Den

[F] = N(F)[E]o/Den

[FB/EQR] = N(FB/EQR)[E]o/Den

[EQ] = N(EQ)[E]o/Den

[ER] = N(ER)[E]o/Den

v(P) = M(P)[E]o/Den

N(E) = k_-1_k_-2_[P] + k_+3_(k_-1_ + k_+2_)[B]

N(EA/FP) = k_+1_k_-2_[A][P] + k_+1_k_+3_[A][B] + k_-2_k_-3_K_4_K_6_[P][Q][R]

N(F) = k_+1_k_+2_[A] + k_-3_K_4_K_6_(k_-1_ + k_+2_)[Q][R]

N(FB/EQR) = k_-1_k_-2_K_4_K_6_[P][Q][R] + k_+3_K_4_K_6_(k_-1_ + k_+2_)[B][Q][R]

N(EQ) = k_-1_k_-2_K_6_[P][Q] + k_+3_K_6_(k_-1_ + k_+2_)[B][Q]

N(ER) = k_-1_k_-2_K_7_[P][R] + k_+3_K_7_(k_-1_ + k_+2_)[B][R]

Den = k_+1_k_+2_[A] + k_-1_k_-2_[P] + k_+3_(k_-1_ + k_+2_)[B] + k_+1_k_-2_[A][P]

k_+1_k_+3_[A][B] + k_-1_k_-2_K_6_[P][Q] + k_+3_K_6_(k_-1_ + k_+2_)[B][Q] + k_-3_K_4_K_6_(k_-1_ + k_+2_)[Q][R] + k_-1_k_-2_K_7_[P][R] + k_+3_K_7_(k_-1_ + k_+2_)[B][R] + k_-2_K_4_K_6_(k_-1_ + k_-3_)[P][Q][R] +

k_+3_K_4_K_6_(k_-1_ + k_+2_)[B][Q][R]

M(P) = k_+1_k_+2_k_+3_[A][B] – k_-1_k_-2_k_-3_K_4_K_6_[P][Q][R]

K_7_K_5_ = K_4_K_6_

K_4_ = k_-4_/k_+4_

K_5_ = k_-5_/k_+5_

K_6_ = k_-7_/k_+7_

K_7_ = k_-7_/k_+7_

**Part 3. Coefficients**

Coef_A_ = k_+1_k_+2_

Coef_P_ = k_-1_k_-2_

Coef_B_ = k_+3_(k_-1_ + k_+2_)

Coef_AP_ = k_+1_k_-2_

Coef_AB_ = k_+1_k_+3_

Coef_PQ_ = k_-1_k_-2_K_6_

Coef_BQ_ = k_+3_K_6_(k_-1_ + k_+2_)

Coef_QR_ = k_-3_K_4_K_6_(k_-1_ + k_+2_)

Coef_PR_ = k_-1_k_-2_K_7_

Coef_BR_ = k_+3_K_7_(k_-1_ + k_+2_)

Coef_PQR_ = k_-2_K_4_K_6_(k_-1_ + k_-3_)

Coef_BQR_ = k_+3_K_4_K_6_(k_-1_ + k_+2_)

**Part 4. Kinetic constants**

num1 = k_+1_k_+2_k_+3_[E]_t_

num2 = k_-1_k_-2_k_-3_K_4_K_6_[E]_t_

K_eq_ = num1/num2

Vmax_f_ = num1/Coef_AB_ = k_+2_[E]_t_

Vmax_r_ = num2/Coef_PQR_ = k_-1_k_-3_[E]_t_/(k_-1_ + k_-3_)

K_mA_ = Coef_B_/Coef_AB_ = (k_-1_ + k_+2_)/k_+1_

K_mB_ = Coef_A_/Coef_AB_ = k_+2_/k_+3_

K_mP_ = Coef_QR_/Coef_PQR_ = k_-3_(k_-1_ + k_+2_)/[k_-2_(k_-1_ + k_-3_)]

K_mQ_ = Coef_PR_/Coef_PQR_ = k_-1_/[K_5_(k_-1_ + k_-3_)]

K_mR_ = Coef_PQ_/Coef_PQR_ = k_-1_/[K_4_(k_-1_ + k_-3_)]

K_ia_ = Coef_P_/Coef_AP_ = k_-1_/k_+1_

K_ib_ = Coef_QR_/Coef_BQR_ = k_-3_/k_+3_

K_ip_ = Coef_A_/Coef_AP_ = k_+2_/k_-2_

K_iq_ = Coef_P_/Coef_PQ_ = Coef_B_/Coef_BQ_ = 1/K_6_ = k_+6_/k_-6_  and Coef_BR_/Coef_BQR_ = K_7_/(K_4_K_6_) =

1/K_5_ = k_+5_/k_-5_

K_ir_ = Coef_P_/Coef_PR_ = Coef_B_/Coef_BR_ = k_+7_/k_-7_ and Coef_BQ_/Coef_BQR_ = 1/K_4_ = k_+4_/k_-4_

**Part 5. Haldane relationship**

Vmax_f_/(Vmax_r_K_eq)_ = K_ia_K_mB_/(K_ip_K_mQ_K_ir_) = K_mA_K_ib_/(K_mP_K_iq_K_ir_) = K_ia_K_mB_/(K_ip_K_iq_K_mR_)

= k_-2_K_4_K_6_(k_-1_ + k_-3_)/(k_+1_k_+3_)

**Part 6. Complete rate equation**

V = (num1[A][B] – num2[P][Q][R])/Den

Den = Coef_A_[A] + Coef_P_[P] + Coef_B_[B] + Coef_AP_[A][P] + Coef_AB_[A][B] + Coef_PQ_[P][Q]

+ CoefBQ[B][Q] + Coef_QR_[Q][R] + Coef_PR_[P][R] + Coef_BR_[B][R] + Coef_PQR_[P][Q][R]

+ Coef_BQR_[B][Q][R]

Multiply numerator and denominator by num2/(Coef_AB_Coef_PQR_) or, equivalently, num1num2/(num1Coef_AB_Coef_PQR_) as needed, where num1num2/(num1Coef_AB_) = Vmax_f_/K_eq_

V = {num1num2[A][B]/(Coef_AB_Coef_PQR_) –

num1num2num2[P][Q][R]/(num1Coef_AB_Coef_PQR_)}/Den =

{Vmax_f_Vmax_r_[A][B] – Vmax_f_Vmax_r_[P][Q][R]/K_eq_}/Den

Substitute kinetic constants for coefficients in denominator, as follows:

Coef_A_: num2Coef_A_/(Coef_AB_Coef_PQR_) = K_mB_Vmax_r_

Coef_B_: num2Coef_B_/(Coef_AB_Coef_PQR_) = Vmax_r_Km_A_

Coef_AB_: num2Coef_AB_/(Coef_AB_Coef_PQR_) = Vmax_r_

Coef_P_: num2Coef_P_/(Coef_AB_Coef_PQR_) = Vmax_r_K_ia_Coef_AP_/(Coef_AB_) =

Vmax_r_K_ia_Coef_A_/(K_ip_Coef_AB_) = Vmax_r_K_ia_K_mB_/K_ip_

Coef_AP_: num2Coef_AP_/(Coef_AB_Coef_PQR_) = num2Coef_A_/(K_ip_Coef_AB_Coef_PQR_) = Vmax_r_K_mB_/K_ip_

Coef_PQ_: num1num2Coef_PQ_/(num1Coef_AB_Coef_PQR_) = Vmax_f_K_mR_/K_eq_

Coef_BQ_: num2Coef_BQ_/(Coef_AB_Coef_PQR_) = num2Coef_B_/(KiqCoef_AB_Coef_PQR_) = Vmax_r_K_mA_/K_iq_

Coef_QR_: num1num2Coef_QR_/(num1Coef_AB_Coef_PQR_) = Vmax_f_K_mP_/K_eq_

Coef_PR_: num1num2Coef_PR_/(num1Coef_AB_Coef_PQR_) = Vmax_f_K_mQ_/K_eq_

Coef_BR_: num2Coef_BR_/(Coef_AB_Coef_PQR_) = Vmax_r_Coef_B_/(Coef_AB_K_ir_) = Vmax_r_K_mA_/K_ir_

Coef_PQR_: num1num2Coef_PQR_/(num1Coef_AB_Coef_PQR_) = Vmax_f_/K_eq_

Coef_BQR_: num1num2Coef_BQR_/(num1Coef_AB_Coef_PQR_) = Vmax_f_Coef_QR_/(K_eq_K_ib_Coef_PQR_) =

Vmax_f_K_mP_/(K_eq_K_ib_)

Den = Vmax_r_K_mB_[A] + Vmax_r_K_mA_[B] + Vmax_r_[A][B] + Vmax_r_K_ia_K_imB_[P]/K_ip_ +

Vmax_r_K_mB_[A][P]/K_ip_ + Vmax_f_K_mR_[P][Q]/K_eq_+ Vmax_r_K_mA_[B][Q]/K_iq_ +

Vmax_f_K_mP_[Q][R]/K_eq_ + Vmax_f_K_mQ_[P][R]/K_eq_ + Vmax_r_K_mA_[B][R]/K_ir_ +

Vmax_f_[P]Q][R]/K_eq_ + Vmax_f_K_mP_[B][Q][R]/(K_eq_K_ib_)

Divide numerator and denominator by Vmax_r_.

Substitute Haldane relationship:

Vmax_f_/(Vmax_r_K_eq_) = K_ia_K_mB_/(K_ip_K_mQ_K_ir_) = K_mA_K_ib_/(K_mP_K_iq_K_ir_) = K_ia_K_mB_/(K_ip_K_iq_K_mR_)

V = {Vmax_f_[A][B] – Vmax_f_[P][Q][R]/K_eq_}/Den

Den = K_mB_[A] + K_mA_[B] + [A][B] + (K_ia_K_mB_/K_ip_)[P] + (K_mb_/K_ip_)[A][P] +

{K_ia_K_mB_/(K_ip_K_iq_)}[P][Q] + (K_mA_/K_iq_)[B][Q] + {K_mA_K_ib_/(K_iq_K_ir_)}[Q][R] + {K_ia_K_mB_/(K_ip_K_ir_)}[P][R] + (K_mA_/K_ir_)[B][R] +

{K_ia_K_mB_/(K_ip_K_mQ_K_ir_)}[P][Q][R] + {K_mA_/(K_iq_K_ir_)}[B][Q][R]

To account for substrate inhibition by B, multiply by (1 + [B]/K_I_) each of the denominator terms that appears in the distribution equation N(E), i.e. K_mA_[B], (K_ia_K_mB_/K_ip_)[P], and {K_ia_K_mB_/(K_ip_K_iq_)}[P][Q], where K_I_ is the dissociation constant for the binding of B to E.

**Part 7. No- and one-product rate equations**

For measurements of initial rates when at least one product is absent, the numerator reduces to

Vmax_f_[A][B].

For measurements of initial rates with no products initially present, the rate equation reduces to:

V = Vmax[A][B]/{K_mB_[A] + K_mA_[B](1 + [B]/K_I_) + [A][B]}

-----------------------------------------------------------------------------------------------------------------

For measurements of initial rates with only product P and substrates present, the denominator reduces to:

Den = K_mB_[A] + K_mA_[B](1 + [B]/K_I_) + [A][B] + (K_ia_K_mB_/K_ip_)[P] (1 + [B]/K_I_) +

(K_mB_/K_ip_)[A][P]

Grouping the [P] terms gives:

Den = K_mB_[A] + K_mA_[B](1 + [B]/K_I_) + [A][B] + (K_ia_K_mB_/K_ip_)[P](1 + [A]/K_ia_ + [B]/K_I_) or

Den = K_mB_[A](1 + [P]/K_ip_) + K_mA_[B](1 + [B]/K_I_) + [A][B] + (K_ia_K_mB_/K_ip_)[P](1 + [B]/K_I_)

The latter arrangement of the equation shows that product P acts in part as a dead-end inhibitor by binding to the enzyme form F, represented in the equation by the term K_mB_[A], in competition with substrate B. The equation also shows that inhibition by product P and substrate inhibition by substrate B are interdependent, such that substrate inhibition by B reduces the effect of product inhibition by P.

-----------------------------------------------------------------------------------------------------------------

For measurements of initial rates with only product Q and substrates present, the denominator reduces to:

Den = K_mB_[A] + K_mA_[B] (1 + [B]/K_I_) + [A][B] + (K_mA_/K_iq_)[B][Q] or

Den = K_mB_[A] + K_mA_[B](1 + [B]/K_I_ + [Q]/K_iq_)+ [A][B]

The latter arrangement of the equation shows that product Q acts as a dead-end inhibitor by binding to the unliganded enzyme form E, represented by the term K_mA_[B], in competition with substrate A.

-----------------------------------------------------------------------------------------------------------------

For measurements of initial rates with only product R and substrates present, the denominator reduces to:

Den = K_mB_[A] + K_mA_[B](1 + [B]/K_I_) + [A][B] + (K_mA_/K_ir_)[B][R] or

Den = K_mB_[A] + K_mA_[B](1 + [B]/K_I_ + [R]/K_ir_) + [A][B]

The latter arrangement of the equation shows that product R acts as a dead-end inhibitor by binding to the unliganded enzyme form E in competition with substrate A.
